# Exploring the value and acceptability of a patient navigator program for people who inject drugs and are hospitalized for bacterial infections: patients’, community organization and healthcare workers’ perspectives

**DOI:** 10.1186/s12879-025-10617-y

**Published:** 2025-02-14

**Authors:** Karine Bédard, Isabelle Boisvert, Marianne Rochette, Eric Racine, Valérie Martel-Laferrière

**Affiliations:** 1 Direction de la qualité, de l’évaluation, de la performance et de l’éthique, Centre hospitalier de l’Université de Montréal, 1000 St-Denis, Montréal, Qc H2X 0C1 Canada; 2 Centre de recherche du Centre hospitalier de l’Université de Montréal, 900 St-Denis, Montréal, Qc H2X 0A9 Canada; 3 Pragmatic Health Ethics Research Unit, Montreal Clinical Research Institute (IRCM), 110 Avenue des Pins Ouest, Montréal, Qc H2W 1R7 Canada; 4 Department of Medicine, Department of Social and Preventive Medicine, Université de Montréal, 2900 Bd Édouard-Montpetit, Montréal, Qc H3T 1J4 Canada; 5 Department of Neurology and Neurosurgery, Department of Medicine (Program in Clinical and Translational Research), McGill University, 845 rue Sherbrooke Ouest, Montréal, Qc H3A 0G4 Canada; 6 Department of Microbiology, Infectious Diseases and Immunology, Université de Montréal, 2900 Bd Édouard-Montpetit, Montréal, Qc H3T 1J4 Canada

**Keywords:** People who inject drugs, Peer navigator, Focus groups, Bacterial infection, Hospitalization

## Abstract

**Background:**

Hospitalizations for bacterial infections are often difficult for people who inject drugs (PWID) and healthcare workers, in part due to biases and stigma associated with substance use, patients’ competing needs, such as pain and withdrawal management, and strict antibiotic treatment protocols. In recent years, peer navigators have been introduced as a strategy to reduce stigma and bridge the gap between patients and healthcare workers, but little is known about their involvement in hospitalization settings. The aim of this study was to assess the value of adding a peer navigator program and to evaluate the elements that key stakeholders identified as essential for the program to be successful.

**Methods:**

This was a qualitative study using focus groups. The interview guide was collaboratively developed by ethicists, physicians, and a person with lived experience and validated with a PWID and a community worker. Three two-hour focus groups were conducted in February 2022 with PWID, community organizations and healthcare workers. Descriptive and interpretive thematic analyses were carried out.

**Results:**

Nineteen people (5 PWID, 6 community organization workers, 8 healthcare workers) participated in the focus groups. The final coding strategy involved 4 main themes: challenges in current care, positive aspects of current care, aspirations for quality care, the contribution of peer navigators as a solution to current challenges and the realization of aspirations. Improvements in the quality of care should focus on an approach centered on patients’ values and aspirations; improving the current hospital environment, particularly in terms of training and communication; and encouraging collaborative partnerships with all parties involved. The integration of peer navigators seems to be a promising strategy for improving communication and trust and, consequently, to facilitate shared decision-making and adapted care.

**Conclusions:**

Our study showed that any innovative model should be centered on patients’ needs and values and therefore co-constructed with them and other parties involved, notably the community organizations offering services to these patients. The inclusion of well-trained and well-supported peer navigators has the potential to improve care and work toward achieving aspirations of quality care.

## Background

Along with the opioid overdose crisis experienced in several countries in recent years, there has been a significant increase in the number of consultations, hospitalizations, and deaths associated with bacterial infections related to injectable drug use, resulting in significant human and financial costs [1–7].

The hospitalization of people who inject drugs (PWID) often presents challenges. The healthcare system can be a stigmatizing environment for them. The reasons for this include biases and stigma associated with substance use, discord between patients’ competing needs (e.g., financial issues, withdrawal, basic needs such as food or shelter) and strict treatment protocols (e.g., isolation in the room, intravenous antibiotic therapy several times a day at set times), and insufficient training for healthcare workers in medical and surgical departments on the specificities of these patients. There is a need among healthcare professionals for training on substance use disorders, concurrent disorders, withdrawal and pain management [8–14]. In addition, PWID often have difficulty navigating the healthcare system, which is commonly experienced as rigid, overwhelming and fragmented [15]. These challenges are often amplified by the presence of mental health issues and social vulnerabilities. This regularly leads to the PWID’s disengagement from their care and delays in consultation and care trajectories, which is characterized by frequent entry and exit in the healthcare system [10, 13, 16, 17]. Among these patients, there is an overrepresentation of patient-directed discharges, and emergency room (ER) reconsultations and readmissions within 90 days [10]. The individual and social impacts of these phenomena, such as more advanced medical conditions at presentation, prolonged or incomplete hospitalizations, and increased mortality are significant [8, 10, 18]. From the perspective of healthcare workers, a combination of conflicting values toward substance use and repeated failures in care episodes can undermine morale and make it seem pointless to invest time and care in a clientele who injects drugs [19]. Ultimately, this can lead to a lack of empathy and disinterest in these patients, which becomes discriminatory and affects the quality of care that the patients receive.

In recent years, navigators have emerged as a solution for reducing stigma and bridging the gap between patients and healthcare workers [15, 20, 21]. Navigators can come from a variety of backgrounds: nurses, community organization workers, people with lived experience, etc. Peer navigators is a term used to refer to people sharing an identity or lived experience with target patients [22]. Peer navigation is a “patient-centered model of care developed in cancer treatment and other areas of healthcare, peer navigators provide guidance and support through complex health systems, acting as a bridge between clinical and community services and social supports” [23]. Tobin et al. defined three components of peer-based approaches: education, social support (emotional, instrumental, and appraisal), and introduction or change of social norm [22].

Although peer navigator approaches have been studied with PWID and have demonstrated convincing benefits [20], they have mainly been used in the context of overdoses, emergency room visits, and HIV/HCV care. However, few studies have been conducted in the context of hospitalization, including infections associated with substance use [15]. Implicating peer navigators within a hospital context is likely to have similar benefits. For example, peer navigators who have experienced hospitalization are more likely to understand the difficulties experienced by patients compared to healthcare workers who tend to have clinical and theoretical knowledge of substance use. This could help establish trust between peer navigators and patients and improve chances of committing to care. It may contribute to adapting existing approaches to better reflect the human and social dimensions of PWIDs’ experiences and their interactions with healthcare teams [24]. However, these objectives can only be achieved if PWIDs perceive the intervention of a third party in their care as positive and if the healthcare teams are open to the integration of peer navigators in an acute care setting, which has not been well documented in literature. The roles of peer navigators can be very broad. When developing a program, identifying the gaps between PWIDs and healthcare workers, or between PWIDs and the healthcare system as a whole, is essential to defining the roles that navigators could play to ensure an appropriate and catered patient-centered program structure.

The institution where the current study took place does not have a peer navigator program for PWID hospitalized for bacterial infections. The aim of this study was to assess the value of adding such a program and to evaluate the elements that key stakeholders identified as essential for a peer navigator program to be successful.

## Methods

This study is based on a qualitative research approach using focus groups. The objectives of this project were to explore the added value and acceptability of peer navigators to improve care. To do this, it was necessary to document current perceived issues and challenges in care as well as aspirations to then evaluate whether peer navigators would alleviate these challenges and fulfill aspirations. The research team relied on the COREQ guideline for reporting qualitative research [25].

The research aim emerged from the numerous clinical ethicist consultation request by the clinical teams, regarding issues related to PWID hospitalizations and quality of care. The orientations that guided the data collection and analysis were drawn from pragmatist and narrative ethics, and from the literature involving peer navigators when working with PWID. Pragmatist and narrative ethics are common and complementary approaches used by clinical ethicists to emphasize dialogue, lived experience, and the consideration of patients’ perspectives as means of identifying, clarifying, and resolving ethical issues that concern them. These approaches emphasize the active role that people with lived experience can play in asserting their experiential perspective and aspirations. Such approaches also emphasize the importance of developing contextually validated interventions that respond to the needs and aspirations of stakeholders [26–30]. These were the main reasons why the research team included a person with lived experience who had been hospitalized for infections related to injection drug use in the past, healthcare workers working with PWID, a clinical ethicist working with both healthcare workers and PWID, and additional ethics researchers.

### Data collection

The interview guide was developed collaboratively by the research team and was divided into three sections: (1) current state of care, (2) areas for improvement and unmet needs, and (3) the peer navigator approach. It was validated with a PWID and a community organization worker, both of whom met the study’s eligibility criteria. Validation of the interview guide was done in person and aimed to test the fluidity and clarity of questions and transitions between sections. This process helped to estimate time for each focus group considering the different stakeholders.

Three two-hour focus groups were conducted in February 2022 with (1) patients with a history of injection drug use and having been previously hospitalized for at least seven days to a bacterial infection, (2) healthcare workers from Centre hospitalier de l’Université de Montréal (CHUM) and (3) community organization workers in Montréal. The focus groups were facilitated by a clinical ethicist (KB) and a person with lived experience (IB) to avoid biases, conflict of interest, and improve participants’ confidence in the exchange of information. The focus groups were audio-recorded to facilitate transcription and to allow the facilitators to be fully focused on the discussion.

To be eligible to participate in the focus groups, PWID had to have a history of hospitalization for a minimum of 7 days for a bacterial infection related to their injection substance use. Efforts were made to include healthcare workers from different professions (nurses, physicians, social workers) and specialties (emergency medicine, internal medicine, addiction medicine). Community organization workers had to work at organizations servicing PWID in the Greater Montreal area. PWID participants were approached through the CHUM’s addiction medicine clinic and word-of-mouth by the person with lived experience using her contact network. She also targeted relevant community organizations and invited them to participate via email. The inclusion of a person with lived experience in the solicitation of PWID participants proved to be essential and helped create a bond of trust in the focus groups. CHUM healthcare workers were contacted by the research team members through their networks and through care unit managers. Brief demographic data was collected from focus group participants.

### Analysis

The recordings were transcribed verbatim by a professional transcription service and verified by KB by listening to the recordings and correcting the transcripts. We followed a template analysis thematic analysis method as proposed by Brooks et al. [31]. Template analysis is a method that provides structure (e.g., it allows for the development of a code book) and flexibility (e.g., no pre-set hierarchies of themes) to thematic analysis. The key steps of this method are: 1. familiarization with the content; 2. preliminary coding of the data; 3. organizing emerging themes into clusters; 4. defining an initial coding template; 5. applying the template to further data with some modifications (as needed); and 6. finalizing the template and applying it to the full data set [31]. The first stage of data familiarization was undertaken by reviewing the verbatim interviews and listening to the audio recordings. For the preliminary coding of the data, an a priori code book based on the main sections of the interview guide (current care, aspirations, and peer navigator program) was developed by KB. This preliminary coding involved parsing out the content into these three themes corresponding to sections of the focus group interview grid. Contrary to other forms of thematic analysis (e.g., Braun and Clarke, 2006), template analysis allows for the use of a priori themes and the development of a code book [31, 32]. KB and IB organized and refined the data into sub-themes and clusters to produce an initial coding template, also revised and discussed with other team members (MR and ER). The subthemes emerged by grouping the content while respecting the three themes (current care, aspirations, and peer navigator program). Subsequently, KB and IB took turns further analyzing the data and refining the template, discussing these revisions with other members of the research team (MR and ER), to eventually apply the template to the final data set, reach a consensus on its application, and ensure that the results and interpretation were aligned with the project’s objectives (see Table 1).

**Table 1:** Overview the template for thematic analysis

Themes	Sub-themes
Current care* (Challenges in current care)	• Characteristics of care environments • PWID characteristics • Relational aspects between PWID and healthcare workers • Withdrawal and pain
Current care (Positive aspects of current care)	• Personalization of care • Efficient patient care
Aspirations for personalized, quality care	• Adapted approach and values • Improvement of hospital services • Partnership system
Peer navigators as a response to challenges and aspirations	• Peer navigator role • Program structure • Profile of the peer navigator • Benefits

* The theme of current care was divided into two themes

MAXQDA (VERBI Software) was used for the preliminary coding phase. Subsequently, a shared Excel spreadsheet was used to facilitate the collaboration between KB and IB to carry out the next steps, refine the template, and facilitate discussions.

For each theme and subtheme, we present a narrative synthesis based on our descriptive and interpretative thematic analysis. The subthemes are presented in the tables of each theme, accompanied by illustrative quotes. In the presentation of the data, participants are anonymized, only their membership in one of the three participant groups is retained: PWID, community organization or healthcare workers.

### Ethical considerations

This research project was approved by the CHUM Research Ethics Committee (21.372). Participants were required to sign an informed consent form prior to engaging in a focus group. The study was conducted in accordance with the principles of the Declaration of Helsinki. Participants were compensated $50 for their participation.

## Results

### Participants

A total of 19 people participated in the focus groups (Table 2). The majority of participants were women and self-identified as White. The healthcare workers group included five nurses, one social worker and two physicians. Community organization workers came from organizations working with people experiencing homelessness, including PWID, offering safe injection equipment and/or working in health promotion for PWID.

**Table 2:** Demographics characteristics of focus groups participants

	PWID, *n* = 5	Healthcare workers, *n* = 8	Community organization workers, *n* = 6
Age, median (range)	45 (33–55)	30.5 (22–43)	34 (25–39)
Gender (women, %)	4 (80%)	8 (100%)	5 (83%)
Ethnicity (%)			
-White	4 (80%)	4 (50%)	5 (83%)
-Indigenous	1 (20%)	-	-
-Black	-	1 (12.5%)	-
-South-East Asia	-	1 (12.5%)	-
-Middle-Eastern	-	2 (25%)	1 (17%)
Profession			
-Nurse		5 (62.5%)	
-Social worker		1 (12.5%)	
-Physician		2 (25%)	
-Service coordinator			3 (50%)
-Project manager			2 (33.3%)
-Community organizer			1 (16.7%)

The final coding strategy had 4 main themes: 1. the challenges of current care, 2. the positive aspects of current care, 3. the aspirations for quality care, and 4. the contribution of peer navigators as a solution to current challenges as well as the realization of aspirations.

### Challenges in current care (Table 3)

**Table 3:** Challenges in current care

Subtheme	Definition	Key content elements	Examples
Characteristics of care environments	Issues, obstacles and challenges in the existing care structure, and difficulties encountered in caring for PWID	• Access to care • Rigid system structure • Continuity of care • Respect for confidentiality • Stigma	“But we get so many closed doors, so many judgments that we don’t even bother to come and ask for help.” (PWID)
PWIDs’ characteristics	Characteristics, character traits and behavioral similarities observed in PWID.	• Craving (urges/obsessions to use) • Great distrust of the healthcare system • Need to go out regularly during the stay, to be autonomous • Need to be listened to and understand given information • Need for love and low self-esteem • Hypersensitivity • Instability • “Right here and right now”	“Then the more our esteem decreases, the less we take care of ourselves, you know, then at a certain point, we don’t even need society judging us anymore, we judge ourselves, you know, then we destroy ourselves.” (PWID)
Relations between PWID and healthcare workers	Difficulties encountered in the therapeutic relationship between healthcare workers and PWID patients	• Bias and prejudice • Lack of knowledge/need for training • Communication	“There’s a great difficulty in expressing the real need for the why-of-the-how of today; what have I got? precisely because they’re in crisis.” (community worker)
Withdrawal and pain management	Issues faced by PWID and healthcare workers in managing withdrawal, pain and opioids agonists treatments	• Lack of knowledge among healthcare workers	“Well, what’s shameful, actually, that I regularly experienced at hospitals during my active consumption, is precisely not to take into consideration that if I tell you "I’m in pain", don’t come and tell me that I want a hit. Don’t think that I’ve come to see you because I’m a drug addict, to get a buzz. I think there’s a way of seeing the difference.” (PWID)

#### Characteristics of care environments

The first issue is access to care. Accessing care through the emergency department is a major issue, accentuated by waiting time and rigidity of the care setting: “You know, showing up in pain as a user, with abscesses, with a physical reality that doesn’t match the punctual reality of their use, waiting 10–11 hours in the hospital, it’s not in their plan for the day” (Community organization worker). Time spent in an emergency department comes in conflict with time spent using, obtaining money needed for using, or carrying out activities that are considered competing priorities for the PWID.

Restrictions related to leaving hospital premises contribute to increased patient-directed discharges, while being accompanied off of hospital grounds is detrimental to patient retention. Discharge planning was a noticeable stage where challenges were reported by participants. At this stage, the lack of communication and coordination between hospital and community organizations are barriers to the continuum of care for patients. This can contribute to rapid and recurrent ER consultations.

### PWID characteristics

All three groups agreed that PWID generally have unfulfilled and urgent needs. The healthcare group and the community organizations group emphasized that these needs are often perceived as immediate by the patient and expressed in the form of distress. This may conflict with the clinical priorities of their treatment plan, as well as with the priorities of other patients who also require care. In particular, PWID have a strong need for autonomy, freedom, and being heard and considered. All three groups reported that characteristics such as hypersensitivity, low self-esteem, and difficulty establishing trusting relationships are common with PWID. Many, if not all PWID have had negative and stigmatizing experiences in health care settings. As a result, they may quickly feel misunderstood or unimportant to healthcare workers, especially when their needs are not met in a delay that they consider acceptable. This leads to further mistrust of health care and social services and feelings of insecurity.

### Relational aspects between PWID and healthcare workers

In all three groups, biases, lack of knowledge, and miscommunication were brought up. Past negative experiences of both PWID and healthcare workers reinforce unconscious biases and prejudices even before clinical management begins. Therefore, the level of communication, listening, and collaboration is critical in determining whether the experience will be positive or negative. However, in hospital settings where there are multiple interactions with healthcare workers (e.g., nurses, physicians, patient attendants, trainees) and frequent rotations of staff, it becomes difficult to ensure consistency of care and of information sharing with patients and among professionals. All groups agreed that good knowledge of the particularities and needs of PWID was essential to improving care. PWID communicated that they had to constantly repeat themselves and that they were often misunderstood. Healthcare workers mentioned that they lacked the knowledge and training to care for PWID properly.

### Withdrawal and pain

As stated by a healthcare worker, “When withdrawal and pain management are not addressed, like, from the start of hospitalization, it really becomes more difficult, even afterwards, to catch up with the therapeutic relationship.” All three groups agreed that assessing withdrawal, relieving its symptoms, and managing pain properly during hospitalizations for bacterial infections were all essential to maintaining the therapeutic relationship. Participants agreed this was where there was the greatest lack of knowledge amongst professionals. This was also reported as a factor that could lead to patient-directed discharge and determine the type of experience from a patient and caregiver perspective.

### Positive aspects of current care (Table 4)

**Table 4:** Positive aspects of current care

Subtheme	Definition	Key content elements	Examples
Efficient patient care	Concrete actions that facilitate proper care of PWID and values that are important to them	• Care plan including basic needs • Dedicated team • Safe care space • Unbiased approach • Support and accompanythem • Communication	“You know, I was going through withdrawal and all that. I end up in a hospital. Finally, they treated me and sent me upstairs. It was great. The nurses were so good. I had food. I had three meals a day, something I really didn’t have on the street. And that’s when I started taking certain steps, not for the last time, but it was part of my steps. In short, you know, like, I got out of there and then I had like a few things I’d done in terms of my health. Then I was like, “I’m leaving better, much better than when I left". And it’s like, it also left me with a great experience, you know. I was like, “Okay, they really took care of me". And yeah, that’s it. I think it can create great experiences too. And different relationships, maybe, with hospitals and all that. If you’re listened to, I think… Yes.” (PWID)
**Personalization of care**	Elements emphasizing the importance of personalizing care for this clientele.	• Patient-centered approach • Unbiased approach • Training • Shared care plan • Culturally safe space • Communication	"When I have one who says: “Listen, I’m sorry, but the doctor’s prescription won’t get you very far”, she goes back to the doc and makes them understand that I have high tolerance. When you have people like that who take the time to understand the state you’re in, what you are going through, and your needs, and don’t just think you want to get high. But that you want to appeased, and then be able to function afterwards. It’s magical when you get staff like that.” (PWID)

#### Personalization of care

The group of PWID emphasized that being cared for by an open-minded person or team with an unbiased, empathetic approach could alter the care experience favorably. Patients want to feel cared for, and they value caregivers who have great listening skills and a certain flexibility that respects the patient’s autonomy in care plans. The PWID and healthcare workers groups expressed more positive care experiences than the community group. The key elements that emerged were training on PWID specificities, non-judgmental care and empathetic communication.

#### Efficient patient care

A clear care plan should be co-constructed with the patient to respect their priorities, such as basic needs (e.g., housing, food) as suggested by one of the healthcare worker “[the patient] has no choice but to be there anyway, so if we can try to help in other ways at the same time, then take advantage of this to try to organize a follow-up, try to make up for something that, in everyday life, they don’t have the time and/or energy to do. Now, there’s a roof over their heads, there’s food, you know, there’s everything they need for basic needs, which can sometimes be difficult to meet, from what I hear, that’s being handled. So, if there’s an opening for other concerns, I think it’s a good time to intervene”. The care plan should be shared quickly following the patient’s hospital admission. This contributes to personalized, safe, and effective care. Likewise, a care setting that respects privacy, especially a private hospital room, was seen as essential by PWIDs.

### Aspirations for personalized, quality care (Table 5)

**Table 5:** Aspirations for personalized, quality care

Subtheme	Definition	Key content elements	Example
**Adapted approach and values**	Values, actions and possible adaptations for effective care of PWID patients	• Start with patient’s needs • Improve first stages of care, i.e., hospital admission • Consistency and continuity of care • Consolidate care • Warm, human care • Training	“To be able to do that, to become aware of my needs, I have to be able to make myself understood, to feel that I belong, to feel that I can, someone will end up reaching out to me. You see, that’s how I see it.” (PWID)
**Improvement of hospital services**	Solutions to existing care structure issues, and concrete adaptation of services offered	• Care trajectory • Support • Training	“This person can also explain to you the steps to come. Because, you know, sometimes, when you don’t know what’s going to happen, it causes anxiety. That’s when, well, you can get triggered, there are lots of other things that can come into play. So, like, having someone explain to you from A to Z: “This is what will happen”. I think that can reduce stress for people so very, very, very much. " (Community worker) "to have someone in-house, as she said, but to help with staff training, and to have a liaison agent at hospital and emergency admission.” (Community worker)
**Partnership system**	Recommendations to better meet the needs of PWID patients before, during and after an episode of hospital care, in a partnership-based approach	• Dedicated and educated team • Mobile care team • Specialized clinic • Ongoing partnership with the addiction service • Care plan	“It would be ideal if there were some kind of partnerships system, particularly with the police, maybe paramedics and the CHUM, or at least Montreal hospitals, to set up a team that could accompany the patient from the police car or ambulance to hospital admission, all the way until hospital release, and keep in touch with community organizations, who, like it or not, are a bit like family to these people.” (Community worker*)*

#### Adapted approach and values

According to all participants, care programs could be improved with an adapted approach centered on values, such as humanity, active listening, safety, and caring. These elements emerged as the pillars of a patient-centered approach for PWID, focused on their needs, and likely to improve the well-being for healthcare workers as well. A community organization worker illustrated this: “Sometimes just realizing that a patient names a need, it’s silly to say, but: ‘I have an abscess. I’d like it to be treated.’ That’s accepting that his lifestyle will continue afterwards. And that’s okay too. If the person wants this, it’s realizing that it’s not always a matter of saying: ‘Well, we’ve got to stop everything, we’ve got to handle this, we’ve got to go through withdrawal’. It’s instead saying, ‘Okay, we’ll treat that.’ [The patient] just wants to make sure there’s no infection”.

All groups agreed that an ideal model of care should consider individual needs of PWID. Granting patients as much freedom and autonomy as possible during their hospitalization and helping them resolve external stressors (e.g., securing housing, cashing a cheque) were identified as key facilitators to adapted and improved care.

#### Improvement of hospital services

With regards to hospital care, participants’ aspirations should be treated by professionals with better knowledge and training on the reality of PWID, to offer a more standardized approach at key moments of the hospitalization and to facilitate PWID treatment completion. Training should be offered to all members of the care team who come in contact with patients, including orderlies.

### Partnership system

Beyond their aspirations in the hospital environment, participants also proposed mobilizing various professionals in an intersectoral partnerships approach (e.g., community organizations, paramedics, police, social services, psychologists, nurses, physicians) to provide proactive, uniform, and improved support and accompaniment for PWID, when necessary, before, during, or after being treated for a bacterial infection in a tertiary hospital setting.

### Peer navigators as a response to challenges and aspirations

Taking into consideration the aforementioned elements, operationalization of a peer navigator program within a hospital context was explored in detail with all groups to identify how it could contribute (or not) to improving care and meeting the needs of PWID and healthcare workers (Table 6).

**Table 6:** Peer navigators as a response to challenges and aspirations

Subtheme	Definition	Key content elements	Example
**Peer navigator role**	Tasks, objectives, responsibilities of a peer navigator	• Vision and objectives • Tasks and responsibilities	“I see him as a resource person to whom, if I am anxious and have specific questions, I can refer to them. A person of reference to whom I can, you know, if I know that I see the doctor only every 72 hours, but it’s good to know that if something happens, this person can get in touch with the doctor and say, ‘Listen, things aren’t going well, you know, is there any way you can stop by to see Mrs. So-and-so or Mr. So-and-so before the planned appointment?” (PWID) “In fact, that’s what it’s all about, it’s acting as a mediator, helping with the transition between this system, the healthcare system, and the real problems of this person who, as I was saying earlier, is a marginalized person, excluded from the system. So, you actually really need a bridge”. (Community worker)
**Program structure**	Important elements and suggestions for the development of a peer navigator program, which clientele should be targeted by this service and the anticipated challenges.	• Targeted clientele • Mandates • Accessibility to peer navigators	Regarding program presentation: “It would be good for an automatic referral for patients who use, have an addiction. So the person would come in automatically to introduce himself. If the person decides not to take this service, that’s their choice, but, you know, the introduction will be done.” (PWID) With regard to the peer navigator’s involvement: “The moment that I find the most favourable, is at the beginning, that’s when I think it’ll be the most useful. Because it’s really the first few days that are a bit harder. There are more professionals coming to see you, there’s more information. I’d tend to want to include them at the beginning.” (Healthcare worker)
**Peer navigator profile**	Valuable qualities, character traits, professional and/or life experience and strengths of a peer navigator to successfully support PWID	• Profiles (experience and training) • Skills required • Barriers to hiring	“someone who has knowledge, but mixed with experience. You know, because sometimes, just the experience, you know, if they say “Yes, yes, you’ll be able to do that”, but in the end, he can’t, you know, because the hospital doesn’t allow it, or you know, it’s out of the system, you know. You know, who knows something about the healthcare system, but has lived through it”. (PWID)
**Benefits**	Benefits of a peer navigator program to support PWID	• Facilitating the discussion of care and treatment management • Reduce isolation • Increase trust • Retention in care	“it’s important that expectations are clearly stated to facilitate the start of hospitalization. Then maybe by addressing, maybe if the patient can address this directly with the peer navigator upon arrival, there are things that could be communicated quicker to the healthcare team, without necessarily having the patient name all of his needs. He doesn’t necessarily arrive with a grocery list either, so…” (Healthcare worker) “With the patient at the beginning, well, sometimes, when I arrive and I don’t know the patient, and then I’m like “What are you using?“, you know, as I ask questions. Sometimes it’s a good thing, because they say to themselves ‘OK, they want to know everything to help me’, but there are others who will be more closed, regardless of if it’s embarrassment or something else, whereas with someone who’s already been through the same experiences, they’ll be more open to them, and we’ll get to the facts quicker." (Healthcare worker)

### Peer navigator role

The peer navigator would act as a spokesperson for both the patients and the healthcare team, facilitating communication between both parties. The peer navigator would assist in explaining the patients’ needs to healthcare workers while clarifying the next steps of the care plan for the patients. This would accelerate the development of a treatment plan specific to the patient’s medical condition and adapted to the priorities and needs of all parties involved, while clarifying the care continuum for patients.

### Program structure

Participants envisioned that accompaniment by peer navigators should be optional and voluntary for patients. This service could be requested by patients or offered to them by the care team at any point during their hospitalization—from admission at the emergency department to discharge from the hospital. Key moments to implicate peer navigators were identified as arrival at the hospital, early stages of hospitalizations, and when preparing for discharge. Involving peer navigators at these stages would improve treatment retention and help facilitate navigation of care for PWID.

Peer navigators should be given ample time to accompany each patient and be easily reachable and available throughout the day. With this in mind, all the groups felt that a peer navigator could not act alone, and they recommended setting up a team. Participants in the PWID group suggested being assigned the same peer navigator during subsequent hospitalizations to ensure continuity of care. An ideal team would be constituted of peer navigators with diverse profiles (i.e., professional background/lived experience, ethno-cultural background, gender identity) and would all have teamwork abilities.

### Profile of the peer navigator

All participants felt that peer navigators should have advanced knowledge on the reality of PWID, either through their own lived experience or professional experience but ideally through a mix of both. Experience with injecting drugs was an important asset, but insight from their experience was necessary. The following quote from a PWID shows how having lived experience with drug use can be conducive to a trusting relationship: “In the best of all possible worlds, you know, it would have to be an addiction counsellor who has already been through it. We have so many, so many workers who have already been through it. You see, there’s a reason why they’re in the addiction field. When someone knows what they’re talking about, and you don’t have to explain yourself in detail, that you don’t even have to explain, it makes it easy to be understood, it’s easier to trust, because it’s like ‘you understand what I’m going through’. So if you understand, and you’re still here, it’s because you want to help me, not because you want to destroy me and put me down. It’s easier to build trust, you know.”

In terms of skills, peer navigators should have the ability to communicate effectively with healthcare teams, including physicians. Good knowledge of the healthcare and community network-including police services, street workers, and community organizations-was considered an important asset to facilitate a continuum of care following hospital discharge. Participants agreed that having good interpersonal skills over technical skills was important for peer navigators.

Community organization workers stressed that peer navigators should be supported in their role, and they should have training to fully understand their role. Ongoing training, particularly in navigation approaches, should be offered. They also stressed the importance of providing personalized accompaniment and psychological support, particularly for peer navigators who might sometimes feel triggered due to their lived experience.

### Benefits

PWID often resist seeking care due to fear of hospitals and fear of judgment from healthcare workers. Participants agreed that being supported by peer navigators would help healthcare professionals better understanding the patients’ needs and help patients better understand needs of the healthcare team, contextualizing both parties’ priorities. Involving a peer navigator would facilitate discussions on treatment plans and help establish trust with the care team. Considering that PWID are regularly stigmatized in hospital settings, the peer navigator could help assert and represent the patient’s rights, ensuring they are taken seriously and treated respectfully.

Participants stressed that the presence and accompaniment of a peer navigator would help reduce isolation experienced by PWID, who may have limited social networks. The peer navigator could support patients, and through sharing their own experiences, might be perceived as an example and inspire patients; giving them hope and motivation to manage their own health.

The community organization workers strongly emphasized that a peer navigator program within a hospital setting would have a positive impact on PWID retention, improve collaboration with healthcare professionals and facilitate completion of antibiotic treatments. Through their front-line experience, they found that accompaniment helped patients navigate care and understand the system better and the continuum of care during hospitalization. Accompaniment could also help reduce stigma and prevent avoidable expulsions and triggers that may lead to patient-directed discharge. This approach could reduce the number of hospital admissions and discharges which are disproportionate amongst this clientele. Ultimately, this could translate into better clinical outcomes and significantly lower costs for the healthcare system. Accompaniment by a peer navigator could potentially reduce patients’ repeated entries and exits from hospital settings which can translate into severe clinical outcomes. This is clearly illustrated by a community organization worker’s testimony: “Then, you know, it would really be a win-win situation because we know when you come in, you go out, you come in, you go out, but then you arrive, and the problem is much bigger. So, you know, a little abscess, it becomes. Listen, we spoke to a doctor the other day, and it was, like, horrible what he told us. A small untreated abscess led to the person being completely paralyzed. But, hey, she could have come in just once. Just once and be well accompanied. Then it’s done, we don’t talk about it anymore. Like, the system would save a lot of money doing a project like that”.

## Discussion

Faced with the increasing prevalence of overdoses and bacterial infections associated with substance use injection, many acute care settings recognize the need to implement innovative models that are better adapted to the needs of their clientele, such as setting up interdisciplinary teams, creating care and service pathways with community organizations, implementing protocols for discharge preparation and management, and integrating harm reduction approaches into clinical care. The peer navigator model is often mentioned in the literature, but has been little explored in the context of physical health care for PWID other than HIV or hepatitis C [15]. It is a complex program to set up, requiring careful planning to ensure that the needs of all parties, including those of peer navigators, are met. The aim of this study was to assess the value of adding a peer navigator program and to evaluate the elements that key stakeholders felt were essential for a program to be successful. Integrating peer navigators into a hospital setting seems to be a promising strategy for improving communication and trust and facilitating shared decision-making and patient-centered care. We highlight how the inclusion of peer navigators responds to current challenges and aspirations related to care for patients, community organizations, and healthcare workers.

### Aiming for a relationship of trust and restoration of PWID’s agency

In recent decades, medical care has evolved from a paternalistic model to a patient-centered approach and now goes beyond by increasingly integrating people with lived experience into care teams [33]. Patients are thus recognized as having valuable experiential knowledge and the ability to co-construct their care plans [34]. However, when working with marginalized groups, such as PWID, we seem to lag behind in adopting such models; some care relationships are marked by a certain infantilization of patients, where healthcare workers decide for them what their priorities should be and what treatments they will or will not be able to adhere to [35, 36]. Our focus groups and literature review clearly demonstrate the importance of respecting individuals’ agency, i.e., their capacity to think and to act, and the value of recognizing this [37–39]. PWID should be invited as partners in their own care, be allowed to make decisions about their own treatment, and set their own priorities. This is not only a desired approach but a fundamental one for a clientele too often accustomed to marginalization and exclusion [40–42]. Respecting individuals and their needs is essential to establishing a trusting relationship [15, 37, 38]. Our study shows that this could be accomplished through the co-construction of a care plan centered around the needs of the PWID [43]. This should serve as a guide for the patient and the treatment team. Focus should include identifying patient priorities, even if they fall outside of the standard medical framework, to help ensure consistency of adapted care.

However, while the benefits of a partnership approach with the patient seem clear, the ways to establish a partnership between the patient and the care team is not straightforward. Similar to what we found in the literature, our study showed that lack of knowledge on the part of both patients and healthcare workers negatively affects care, leading to conflicts of values and priority management [10, 15, 38, 44]. Patients’ lack of understanding of the structure and what is required by the healthcare system may generate anxiety and frustration. Some of them may feel overwhelmed, while others, feel stigmatized. Lack of understanding by healthcare workers of the social and medical realities of PWID puts them at particular risk of offering suboptimal care and services. This is particularly true for pain and withdrawal management [10, 37, 45, 46]. This can have repercussions on the patient’s physical health and can be perceived by patients as a manifestation of stigmatization or lack of listening from healthcare workers, which creates more tension and amplifies misunderstanding between parties [10, 41]. Poor pain and withdrawal management are among the main causes of frustration between PWID and their care team, and patient-directed discharge [10, 14]. In many institutions including at the CHUM, access to addiction medicine and pain management consultation services is not immediate upon patient arrival, and is only provided at the request of the attending physician. Additionally, patients who wish to continue using drugs might refuse addiction medicine referral. Again, this highlights the importance of providing training to all healthcare workers who may work with PWIDs, in order to help health professionals quickly recognize and manage specific medical needs. This would facilitate rapid pain and withdrawal management.

### Peer navigators as facilitators of partnership relations

As seen in the literature, our study suggested that peer navigators may act as a bridge between patients and healthcare workers and between hospitals and the community [15, 38]. They can thus serve as facilitators in communicating all parties’ needs and be advocates for patients which could help restore trust with healthcare workers [15, 38]. Our study showed that a peer navigator could also facilitate this by assisting with dialogue between patients and healthcare professionals and promote respect, listening, safety, and autonomy, which were all needs expressed by PWID participants. As the PWID’s spokesperson in moments of great vulnerability, peer navigators could enable them to regain their place in the discussions and decision-making that concerns them. In fact, the ability to understand and be informed about care and to actively participate in one’s own care are two important dimensions of individual fulfillment that [47] are fostered by a peer navigator approach.

Likewise, creating a culturally safe space for patients is crucial to optimizing their care experience [14]. As one of the roles of peer navigators is to re-establish trust in the system, it is likely that a peer navigator who can be identified to the same minority group as the patient’s could ease this process. Our study underlines the importance of having a team of peer navigators with diversified experiential knowledge who are well trained and well supported in their training needs. In addition, the importance of peer training and supervision is a point that emerges in the literature [15]. Supporting peers in their work is important, as this kind of work can be psychologically demanding and sometimes triggering.

### Planning the evaluation of a future program

While not addressing all the needs identified by our focus groups, the peer navigator model can address several of the discussed challenges and aspirations. These include the development of a safe environment, trusting relationships, and a mutual understanding of needs. In addition to the classic quantitative outcomes (patient-directed discharge, reconsultation/readmission, completion of antibiotic treatment, and mortality), our study suggests that evaluation of a future patient navigator program should assess the quality of withdrawal management, improvements in communication, destigmatisation of substance use and PWID, and more broadly, caregiver satisfaction with episodes of care. This last point could be assessed by comparing individuals’ expectations, needs, and priorities toward the healthcare system and evaluating their level of trust in it, including whether they experience less identity shock upon entering it, and whether they feel more empowered [48, 49].

### Strengths and limitations

The greatest strength of our study is its collaborative research design, which included researchers from diverse backgrounds: physicians, ethicists, and people with lived experience with substance use injection. Our principal person with lived experience (IB) played a leading role in the study, including in questionnaire development, recruitment, interview facilitation, results analysis, and manuscript development.

The results of our study have several limitations. First, they reflect experienced issues and perspectives but do not represent the results of a prospective study. Nevertheless, we present practical findings on how a team of peer navigators might be involved in a hospital setting for health issues for PWID. Second, our sample potentially lacks the representation of certain groups. Notably, it includes a large majority of women, a common bias in qualitative studies [50]. For healthcare workers, this represents the reality of care, with almost exclusively female nursing staff and an increasing feminization of medical practice in Canada [51]. For patients, however, this does not reflect the predominance of men among people who inject drugs. By way of comparison, in Quebec, 75.5% of participants in the SurvUDI study-an epidemiological surveillance network for PWID-were men [52]. Finally, this project took place in a tertiary care hospital that receives many PWID and includes an addiction medicine department that is very active in clinical care and research. The results of our study may therefore be less generalizable to settings not benefiting from such services or caring for a smaller PWID population.

## Conclusions

Beyond the issues directly related to health problems, hospitalization for a bacterial infection remains a potentially traumatic event for PWID. Our study showed that any innovative model must be centered on patients’ needs and values and co-constructed with them and other players involved, notably the community organizations offering services to these patients. Each must be aware of the other’s needs and realities and of what is important to them. For example, if PWID are to be involved in their care and for it to be meaningful to them, healthcare workers must understand and focus on each patient’s needs, including withdrawal and pain management. This requires knowledge mobilization and training of healthcare workers. Therefore, improvements in the quality of care should include an approach centered on patients’ values and aspirations; improving the current hospital environment, particularly in terms of training and communication; and adopting a partnership approach with all involved parties. The inclusion of well-trained and well-supported peer navigators has the potential to improve care and achieve these goals.

## Data Availability

The datasets generated during the current study are not publicly available due participants’ personal information available in the verbatim, but are available from the corresponding author on reasonable request. In that situation, the authors will then ensure that all identifying information is removed.

## Abbreviations

PWID: People who inject drugs
ER: Emergency room
CHUM: Centre hospitalier de l’Université de Montréal
